# Deep Conservation of Human Protein Tandem Repeats within the Eukaryotes

**DOI:** 10.1093/molbev/msu062

**Published:** 2014-02-03

**Authors:** Elke Schaper, Olivier Gascuel, Maria Anisimova

**Affiliations:** ^1^Department of Computer Science, ETH Zürich, Zürich, Switzerland; ^2^Institute of Integrative Biology, Department of Environmental Systems Science ETH Zürich, Zürich, Switzerland; ^3^Institut de Biologie Computationnelle, LIRMM, CNRS–Université Montpellier, France; ^4^Swiss Institute for Bioinformatics (SIB), Lausanne, Switzerland; ^5^Present address: Institute of Applied Simulation (IAS), School of Life Sciences and Facility Management, Zürich University of Applied Sciences (ZHAW), Wädenswil, Switzerland

**Keywords:** protein evolution, tandem repeats, conservation, phylogenetic analysis

## Abstract

Tandem repeats (TRs) are a major element of protein sequences in all domains of life. They are particularly abundant in mammals, where by conservative estimates one in three proteins contain a TR. High generation-scale duplication and deletion rates were reported for nucleic TR units. However, it is not known whether protein TR units can also be frequently lost or gained providing a source of variation for rapid adaptation of protein function, or alternatively, tend to have conserved TR unit configurations over long evolutionary times. To obtain a systematic picture, we performed a proteome-wide analysis of the mode of evolution for human protein TRs. For this purpose, we propose a novel method for the detection of orthologous TRs based on circular profile hidden Markov models. For all detected TRs, we reconstructed bispecies TR unit phylogenies across 61 eukaryotes ranging from human to yeast. Moreover, we performed additional analyses to correlate functional and structural annotations of human TRs with their mode of evolution. Surprisingly, we find that the vast majority of human TRs are ancient, with TR unit number and order preserved intact since distant speciation events. For example, ≥61% of all human TRs have been strongly conserved at least since the root of all mammals, approximately 300 Ma. Further, we find no human protein TR that shows evidence for strong recent duplications and deletions. The results are in contrast to the high generation-scale mutability of nucleic TRs. Presumably, most protein TRs fold into stable and conserved structures that are indispensable for the function of the TR-containing protein. All of our data and results are available for download from http://www.atgc-montpellier.fr/TRE.

## Introduction

Tandem repeats (TRs) are sequence repetitions that occur right next to each other. They are typically classified by the length of the tandemly repeated unit into: microsatellites (<10 bp), minisatellites (10–100 bp), and, albeit less widely used, megasatellites (>100 bp) (Gondo et al. 1998; Thierry et al. 2010). Nucleic TRs are often found to be polymorphic in terms of the number of TR units as a result of high unit gain/loss rates (Gondo et al. 1998; Vergnaud 2000; Bhargava and Fuentes 2010), though these rates are highly heterogeneous among TR loci.

Protein TRs are a major element of protein sequences in all domains of life. They are typically encoded by nucleic TRs in coding regions such that a TR unit duplication/loss generally does not involve a frameshift (Toth 2000). However, protein TR regions may span multiple exons. Protein TRs exhibit a high sequence diversity (Marcotte et al. 1999; Schaper et al. 2012), reflected in an equally high structural diversity (Kajava 2012). For instance, protein TRs may fold into fibrous structures, solenoids, or, for tandem repetitions of whole domains, “beads on a string” organizations (Kajava 2012). On the other hand, protein TRs are also often associated with unstructured regions (Tompa 2003; Simon and Hancock 2009; Jorda et al. 2010; Szalkowski and Anisimova 2011).

The high gain/loss rates of nucleic TRs were proposed to be suggestive of comparable high rates for protein TRs (Tompa 2003). In particular for micro- and minisatellites, examples of highly variable protein TRs were observed in all domains of life (MacDonald 1993; Sawyer et al. 1997; Coil et al. 2008; Butler et al. 2009; Chevanne et al. 2010; see Gemayel et al. 2010; Riegler et al. 2012). Changes in protein TRs typically affect the protein sequence and the eventual folding and functionality of the protein product, leading to changes in phenotype or fitness. For example in human, the expansion of polyQ tracts in *Huntingtin* leads to Huntington's disease (MacDonald 1993; TR unit length *l* = 1), while in fruit flies, polyQ variation in the per gene tunes its circadian clock (Sawyer et al. 1997). Other examples were observed for minisatellite TRs: An association of TR unit gains/losses with phenotypic traits was found in *Saccharomyces cerevisiae*, where variation in flocculin TRs (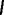 ∼ 45 amino acids or aa) resulted in modulated cell adhesion properties (Verstrepen et al. 2005); a similar association was proposed for several *Candida albicans* genes (Butler et al. 2009; e.g., *l* ∼ 32 aa).

It has been argued that variation in functional TRs may provide a source of genetic variability allowing for fast adaptation (Marcotte et al. 1999; Levdansky et al. 2007; Richard et al. 2008; Chevanne et al. 2010; Riegler et al. 2012), for example, in an evolutionary arms race. Conversely, conservation of TRs may also be an indicator of functional relevance. However overall, little is known at current about the evolutionary modes and time scales of protein TRs.

To study their evolution, the sequence similarity of TR units within a single TR may be analyzed to infer gain/loss events (Björklund et al. 2006, 2010; Light et al. 2012). For example, Björklund et al. (2006) used patterns of TR unit similarity to show that the duplication of multiple units is more frequent than the duplication of single units for common tandemly repeated protein domains. This approach is implicitly based on the assumption that the TR units evolved from a common ancestral unit and therefore can be described by a TR unit phylogeny.

For the related problem of gene paralogs arranged in tandem, gene phylogenies (also called duplication histories in this context) were shown useful for systematic studies of gene gain/loss events (Elemento et al. 2002; Lajoie et al. 2007). Here, we use a similar idea to study the evolution of TR units. Compared with gene phylogenies, the analysis of TR phylogenies is challenging due to shorter unit lengths introducing errors in the reconstructed phylogenies. Yet, here we show that TR phylogenies display clear patterns that shed light on their evolution.

**Fig. 1.**
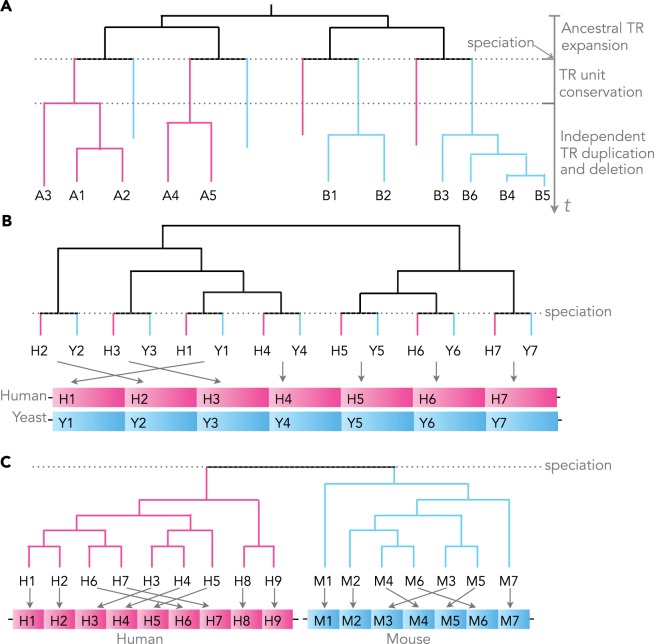
Tandem repeat unit evolution. (*A*) A scenario of TR unit evolution for species *A* and *B* represented by TR unit phylogeny, where nodes mark either speciation events or TR unit duplications. Abandoned edges mark a TR unit loss. The ancestral TR region is created through duplications of an ancestral subsequence, that is, the unique TR unit at the root of the phylogeny (black). Immediately following the speciation event, exact copies of the TR reside in orthologous proteins in both species (pink and blue), even after some point mutations in TR units the TR still is perfectly conserved, as long as the amino acid identity remains high. The 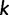
th TR unit in *A* is the closest to the 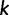
th unit in *B*. Subsequent TR unit duplications and losses diminish the conservation of the TR between species *A* and *B*. Without point mutations, the more TR unit losses or gains occur, the more TR units begin to cluster by sequence similarity within the same species. (*B*) The bi-species TR unit phylogeny of a perfectly conserved WD repeat (PF00400) in the human TORC subunit ENSP00000457870 and its yeast ortholog YNL006W. The TR units are indexed by their order along the protein sequence. The depicted phylogeny allows to reconstruct ancient TR unit duplications leading to the currently observed TR regions in fungi and animals before their divergence ∼0.6–1.6 byr ago (Taylor and Berbee 2007). (C) The bi-species TR unit phylogeny of a perfectly separated TR in the human NAC-alpha domain-containing protein 1 ENSP00000420477 and its mouse ortholog ENSMUSP00000049490. The ancestral protein presumably contained a TR region with multiple repeat units. Yet, the TR region cannot be reconstructed due to the fast succession of TR unit gains/losses in at least one of the lineages.

Any observed TR region may have been conserved over a long evolutionary time scale, or alternatively, it may have originated very recently through rapid evolution. Without the study of orthologous TR regions from multiple species, it is impossible to deduce whether a TR is of a recent or ancient origin. To shed light on the age of a TR, one requires a mapping of TR unit gains/losses to speciation events, based on alignments of orthologous proteins with TRs from multiple species. This approach has been used to study the evolution of TR regions according to the TR unit number, similar to the analysis of microsatellite DNA data (see Bhargava and Fuentes 2010 for a review). Here, we expand this idea to conduct a systematic phylogenetic analysis of TR units from multiple species to test whether TR regions evolve rapidly or are predominantly conserved. Both rapid and conserved modes of evolution may uncover different functional categories of proteins, where the evolution of TRs is presumably shaped by their contribution to the function of the whole protein. For pairs of species, we build TR unit phylogenies including all TR units from the orthologous proteins in both species. The resulting “bi-species” TR unit phylogenies reflect both the ancestral expansion of the TR units before speciation, and lineage-specific gains/losses after speciation (fig. 1*A*). Thus, this approach allows us to recognize TRs that have been conserved since the speciation event. For some proteins, such as the human TORC subunit LST8 (fig. 1*B*), the conservation can be traced back deep to the common ancestor of human and yeast. On the other hand, we are also able to identify TRs that have been subject to recent TR unit gains/losses (fig. 1*C*). Note that besides TR units gains/losses, other mechanisms such as intragenic conversion may lead to modifications in the TR configuration (i.e., number and order of TR units). An intragenic conversion event may be approximated by a TR unit loss that is immediately followed by a TR unit gain. When referring to gains/losses, we implicitly include such alternative mechanisms in the remainder of this article.

In the following, we describe the multispecies analysis of TR unit phylogenies in detail, and introduce criteria to classify TRs according to their mode of evolution. We apply this approach in a proteome wide study on the evolution of human TRs with TR unit length ≥15 aa in comparison with orthologous TRs in 60 other eukaryotic species. For the vast majority of these human TRs, our analyses reveal a surprisingly sustained conservation of TR unit configurations (i.e., number and order of TR units) throughout the eukaryotic kingdom. Finally, we correlate the results with structural features of TRs, as well as functional annotations of TR-containing proteins, to better understand the origin and consequences of the different evolutionary modes. All material and data are available for download at http://www.atgc-montpellier.fr/TRE (last accessed February 20, 2014).

## New Approaches

To analyze the evolution of human TRs across the eukaryotic clade, we developed a phylogenetic pipeline, which can be summarized as follows:

TRs were annotated exhaustively in the human proteome using specifically devised circular profile hidden Markov models (cpHMMs).

1. For each human TR-containing protein, orthology annotations were obtained from the complete proteomes of 60 other eukaryotic species from Ensembl Compara (Vilella et al. 2009; Flicek et al. 2012). For each annotated human TR, homologous TR regions in all orthologous proteins were searched using the corresponding cpHMM.
2. The selected 60 species considered in our study are separated from the human lineage by a wide range of divergences, from chimp (the closest) and mouse, to zebrafish and baker’s yeast (the furthest). Comparisons of TRs in orthologous proteins in all pairs of species allowed us to describe the TR evolution since the speciation events of the species pairs. Taken together, these bispecies comparisons permitted to backtrack the conservation of human TRs throughout the eukaryotic clade. For this purpose, we reconstructed bispecies TR unit phylogenies for all pairs of TR-containing orthologs (fig. 1). These phylogenies were evaluated to ascertain whether the human TR unit configurations have been “conserved” since speciation (fig. 1*B*), or alternatively, became “separated” through gains or losses of TR units after speciation (fig. 1*C*).
3. The classification of TRs according to conserved and separated modes of evolution was correlated with functional annotations and other TR characteristics, notably the type of Pfam family.

### Annotation of Tandem Repeats in the Human Proteome

**Fig. 2.**
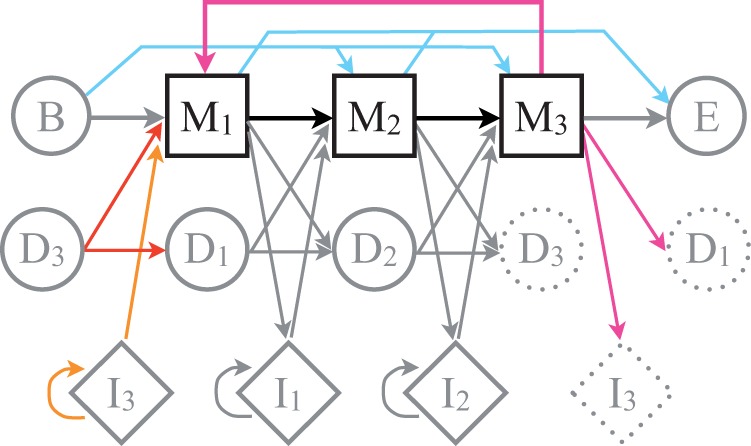
Circular TR sequence profile HMM. Shown is an example of a profile HMM describing a TR unit with three consensus positions, where basic match states (M), deletion states (D), insertions states (I), and transitions correspond to the HMMER core model (Eddy 2008). Repetitions of the motif in tandem are modeled by introducing transitions from the final consensus position to the first consensus position. The transitions probabilities for the final match state (pink), deletion state (red), and insertion state (orange) are taken as the normalized means of the corresponding transitions probabilities in all other consensus positions. The probability to enter the TR is equal for all match states (blue). Similarly, for all match states it is assumed to be equally likely to stay in the TR or leave the TR (blue).

To annotate consecutive repeat units in sequence data, we have developed cpHMMs based on a sequence profile of a potential TR unit. In our model, the match states describe the consensus positions of the TR unit (fig. 2). In contrast to the standard profile HMM (Eddy 1998), we introduced transitions between the last and the first match states, so that one TR unit could be directly followed by the next, until no further match is found (pink, orange, and red transitions in fig. 2). This way, TR regions with an arbitrary number of units can be inferred. Furthermore, as TR units often do not have well-defined boundaries (Schaper et al. 2012; Szalkowski and Anisimova 2013), we assumed that a new TR was equally likely to start and end at any of the positions of a profile (blue transitions).

A set of potential TR unit profiles was obtained from known protein domains and de novo detection of TRs in the human proteome. As the Pfam database (Punta et al. 2011; Mistry et al. 2013) includes many prominent protein domains found in tandem, we used Pfam annotations to look for potential TRs. Currently, ∼40% of the human proteomic sequence carry Pfam-A annotations. For all Pfam-A annotations, we constructed cpHMMs, allowing the possibility that any Pfam-A domain might be repeated in tandem. As short and rare TRs are not expected to be part of the Pfam database, we additionally built cpHMMs from de novo TR detections. Because of algorithmic differences, the TR detection by existing de novo TR detection algorithms is highly disparate (Schaper et al. 2012). Thus, rather than using any single algorithm, we combined the TR predictions of four available algorithms (Materials and Methods).

Finally, the cpHMMs were used to annotate human TRs. To control the number of false positive TRs, each annotation was statistically validated using a model-based likelihood ratio test (Schaper et al. 2012). In summary, we obtained a set of human TRs and corresponding cpHMMs.

### Annotation of Orthologous Tandem Repeats in Eukaryotic Species

To study the evolution of human protein TRs, we compared them with homologous TRs in orthologous proteins of eukaryotic genomes. Orthology annotations and protein-wide sequence data for 60 other eukaryotic species were obtained from Ensembl Compara (Vilella et al. 2009; Flicek et al. 2012). For each human TR, we applied the corresponding cpHMMs across the set of orthologous proteins to detect all homologous TRs.

### Using Phylogenetic Patterns to Study TR Evolution

To systematically determine the mode of TR evolution, we studied bispecies TR unit phylogenies, assuming that all units have descended from an ancestral unit. In particular, for all human versus nonhuman orthologous protein pairs in our data set, we reconstructed phylogenetic histories of TR units similar to those in figure 1. Compared with multispecies TR unit phylogenies, bispecies phylogenies are simpler to analyze, whilst containing sufficient information about the TR evolution in the human lineage. To ensure accurate phylogeny reconstruction, the TR units need to be informative about their gain/loss history. Therefore, we excluded from our analysis all short TRs with unit length less than 15 as well as all TRs with less than 4 TR units.

Reconstructed bispecies TR unit phylogenies helped us to determine whether TR unit duplications occurred before or after the speciation events separating both studied species. This permitted to classify the TR unit phylogenies according to different modes of evolution: long-standing TR unit conservation, and recent TR unit separation (fig. 1). To analyze the phylogenetic patterns of bi-species TR unit phylogeny from species *A* and *B*, we calculated the following statistics:

- The TR unit numbers in the two orthologous proteins (*A* and *B*), 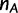 and 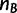;
- The number of cherries (i.e., pairs of leaves that share the parent node on the phylogeny; McKenzie and Steel 2000), 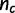, and the number of bispecies cherries, that is, formed by TR units of both species *A* and *B*, 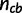. For example, H2 and Y2 in figure 1*B* form a bispecies cherry, and it is 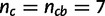 for the entire phylogeny.
- The conservation of TR unit order in bispecies cherries, measured by the Kendall rank statistic 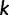 computed on the pairs of indices representing TR unit order. More precisely, TR units from the two species *A* and *B* were indexed by their order in the protein sequence from 1 to 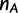 or to 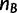, respectively. This way, a pair of order indices was recorded for each bispecies cherry. For example, a bispecies cherry formed by the first TR unit in one species and the third unit in the other species has an index pair of 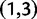. If each of species *A* and *B* contain four TR units and their order is perfectly preserved, the index pairs will be (1,1), (2,2), (3,3), and (4,4) leading to 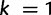. For example, it is 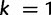 for the phylogeny in figure 1*B*.
- The parsimony score 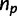 computed over the TR unit phylogeny on species labels *A* and *B* (Felsenstein 1988; Steel 1993). The parsimony score is equivalent to the number of splits on the phylogeny that are necessary to separate all TR units of species *A* from all TR units of species *B*, and is thus a measure for the separation of these TR units through gains and losses. For example, 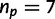 for the example phylogeny in figure 1*B*, where the speciation of *A* and *B* happened after the duplication of an ancestral TR unit in the ancestor of *A* and *B*. In contrast, 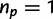 for the example in figure 1*C*, indicating that the speciation event was followed by TR unit gains and losses in at least one lineage.

In the next subsections, we describe simple classification rules computed from these statistics to distinguish between TRs with conserved or separated TR configurations.

### Detecting Protein Tandem Repeat Conservation

In one possible evolutionary scenario, no unit duplications or losses occurred in the TR region in neither lineage of two species after speciation: The ancestral TR unit configuration (i.e., number and order of TR units) is then fully preserved in the current day species. Directly after the speciation, the closest relative of any TR unit in the ancestral protein of species *A* is the (homologous) TR unit in the orthologous ancestral protein of species *B* (fig. 1*A*). If no TR unit gains or losses occurred since speciation (fig. 1*B*), the order and the number of TR units remains the same and the TR unit phylogenies fulfill 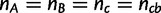 and 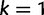. We call such TRs *perfectly conserved* between species *A* and *B*. Presumably, the numbers of “perfectly conserved” TRs would be underestimated in our analysis, mostly due to errors in phylogenetic reconstruction, and in orthology annotation (see Materials and Methods for details). To cushion errors in TR annotation and phylogeny reconstruction, we attributed *strong TR unit conservation*, if 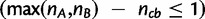, 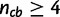, and 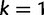. In comparison with “perfect conservation,” this classification rule allows that one TR unit may not have been detected, or that the *i*th TR unit in one species may not pair with the *i*th TR unit in the second species in one case (see examples of “strongly conserved” TR phylogenies in supplementary fig. S2, Supplementary Material online).

The false-positive annotation of strong or perfect conservation is unlikely. For example, the probability of falsely assigning perfect conservation to a pair of random TRs with *n* TR units is 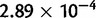 for *n* = 4, and as low as 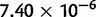 for *n* = 5 (see Materials and Methods for the derivation, and table 2 for *P* values for 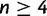). Thus, bispecies TR unit phylogenies displaying perfect conservation indicate that no TR unit gains or losses are likely to have occurred in either lineage since speciation. In comparison, the probability of assigning strong conservation by chance is higher with 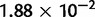 for *n* = 4 and 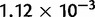 for *n* = 5, albeit still sufficiently small for our attenuated measure to be reliable.

**Table 2.** Probability of Assigning *Perfect Conservation (**Eq. 1**), Strong Conservation (**Eq. 4**)* or *Perfect Separation (**Eq. 5**), Strong Separation (**Eq. 6**)* to a Pair of Random TRs with 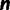 TR Units Each.

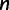	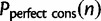	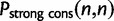	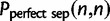	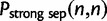
4	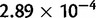	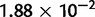	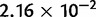	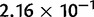
5	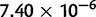	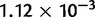	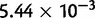	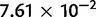
6	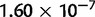	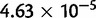	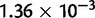	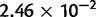
7	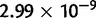	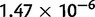	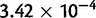	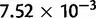
8	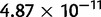	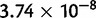	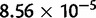	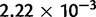

Based on these definitions, we used multiple bispecies comparisons to backtrack the conservation of human TRs. This way, we can ascertain the conservation of a human TR up to the speciation time of human and the species most distant to human, for which perfect conservation of the TR was still observed. This speciation time should be considered as a lower boundary to the estimate of the actual time since when a human TR had been conserved. Note that we do not necessarily find the TR to be conserved among all other descendants since this speciation node. TR conservation may be obfuscated in some descendants due to errors in orthology annotation and phylogeny reconstruction. Also, some of the other descendent lineages might be subject to a different mode of TR evolution, with frequent unit gains and losses.

### Detecting Protein Tandem Repeat Separation

The frequency of TR conservation in human proteome was contrasted with the frequency of TR separation, whereby all TR units from one species were more closely related to each other than to any TR unit in the other species, and vice versa. TR separation was evaluated based on the following rules. Two homologous TRs were assumed to exhibit “perfect TR unit separation” if 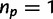 (cf. fig. 1*C*). As discussed earlier, due to errors in tree reconstruction, TR pairs that are “perfectly separated” in reality might not appear separated into two clades on the inferred unit phylogeny. To account for some of these cases, we introduced the following relaxed condition: a pair of TRs exhibited “strong separation” when 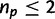. In this case, the bispecies TR unit phylogeny can be partitioned into two or three monophyletic clades (see examples of “strongly separated” TR phylogenies in supplementary fig. S3, Supplementary Material online).

In a more complex scenario, some TR units of the same TR region may be conserved, whereas others may undergo duplications and losses. To account for this case, we attributed “difference in TR unit number” to pairs of TRs with unit number difference ≥4.

Among others, the analysis of TR unit separation between closely related species allowed us to identify TRs that have undergone gains/losses recently. Potentially, such TRs might be subject to ongoing TR unit number changes. Note that errors in orthology annotation might lead to an overestimation of the numbers of separated TRs. On the other hand, overestimations due to phylogeny reconstruction errors are less likely. For example, the probability of falsely assigning “perfect separation” (strong separation) to a pair of random TRs with *n* TR units is 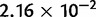 (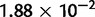) for *n* = 4 and 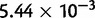 (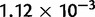) for *n* = 5 (see Materials and Methods for details, and table 2 for *P* values for 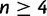).

## Results

### Distribution of TRs in Human Proteins and Their Eukaryotic Orthologs

We detected 3,091 nonoverlapping TRs (with ≥4 TR units of length 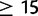) in 2,532 (13%) of all 20,162 human proteins. Of all detected TRs, 356 were de novo annotations, 570 were zinc finger repeats, 225 were leucine-rich repeats (LRRs), and 186 were WD40 repeats (table 1). In total, 193 different PFAM-A domains were found as repeated in tandem in at least one human protein. In the following, we refer to different TRs as of the same type if they were detected with the same cpHMM profile (describing either one of the 193 PFAM-A domains or the 356 distinct de novo detected TRs). The observed distribution of annotated TRs among the TR types was highly uneven, with 43% (70%) of all TRs described by just 1% (5%) of all TR types.

**Table 1.** TR Summary.

	All TRs	Zn Finger	LRR	WD40	ANK	Cadherin	I-Set	De Novo
Human								
Count	3,091	570	225	186	166	107	72	356
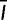	48.5	27.9	24.0	42.2	33.2	104.8	90.9	28.4
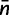	8.8	11.2	10.7	6.8	7.7	6.9	7.8	8.0
SD 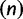	8.2	5.6	5.6	3.6	4.9	5.8	9.2	7.0
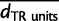	0.96	0.50	1.14	1.35	1.03	1.13	1.30	0.64
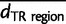	0.4	0.5	0.2	0.1	0.1	0.2	0.2	1.4
PFAM ID	—	PF00096	PF00560	PF00400	PF00023	PF00028	PF07679	—
Other eukaryotes								
Chimp	2,718	502	209	163	155	78	67	308
*p.c.*	2134	379	164	145	134	62	55	177
*c.s.*	0	0	0	0	0	0	0	0
Mouse	1,935	643	121	96	72	59	38	138
*p.c.*	833	86	46	71	49	49	27	47
*c.s.*	91	85	0	0	0	0	0	5
Rat	1,485	289	117	80	69	68	37	127
*p.c.*	751	81	40	57	44	55	30	40
*c.s.*	34	28	0	0	0	0	0	5
Xenopus	1,882	110	153	126	136	166	46	82
*p.c.*	820	50	24	66	49	152	20	19
*c.s.*	16	11	0	0	0	0	4	1
Fugu	2,074	162	215	157	150	95	68	112
*p.c.*	808	56	29	81	54	62	44	22
*c.s.*	42	39	0	0	0	0	0	0
Zebrafish	2,991	484	243	158	166	378	88	127
*p.c.*	1,198	59	34	93	65	351	46	26
*c.s.*	293	283	0	0	0	0	0	8
Fruitfly	1,221	129	174	128	79	18	45	45
*p.c.*	203	15	2	50	11	2	0	4
*c.s.*	36	25	0	0	0	0	0	5
Roundworm	722	21	54	72	63	17	28	78
*p.c.*	72	6	0	18	5	0	0	0
*c.s.*	14	1	0	0	0	0	0	2
Yeast	276	2	15	88	12	0	0	17
*p.c.*	33	0	0	14	1	0	0	0
*c.s.*	5	0	0	0	0	0	0	1

Note.—Characteristics for all analyzed human TRs, averaged over the six most frequent TR types, and de novo annotated TRs: 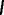 denotes the TR unit length, 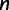 the number of TR units per TR, 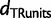 the ML estimate of substitution rates per site separating the TR units within one TR, 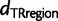 the ML estimate of substitution rates per site separating the TR regions in human and mouse (Materials and Methods). The total number of orthologous TRs for selected eukaryotic species. *p.c.* and *c.s.* are the numbers of perfectly conserved and perfectly separated TRs, respectively. For every TR, we derived the probability of falling into either of these categories by random chance (Materials and Methods). The underlined values indicate that many more cases were found than expected by random chance (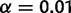).

**Fig. 3.**
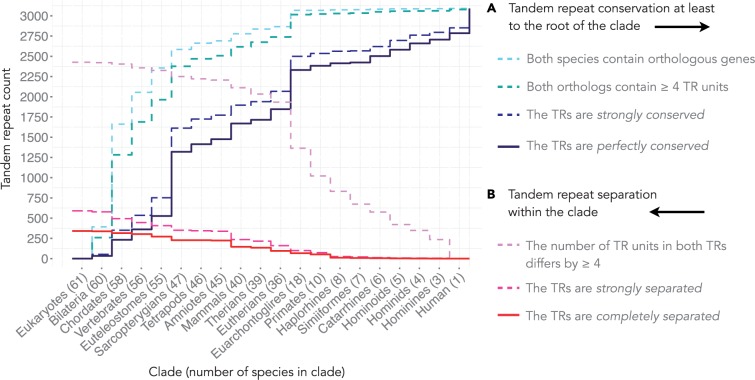
Conservation and separation of 3,091 human protein Tandem repeats (TRs) across the eukaryotes. (*A*) The *y*-axis shows the number of human TRs conserved at least since the root of different reference clades denoted on the *x*-axis and ordered by their generality. We established conservation in a cross-comparative analysis of human TRs with their orthologous TRs in all species outside the clade. Denoted in blue are the four different measures of sequence conservation, where darker color marks a higher degree of conservation. To establish conservation of a human TR at least to the root of a clade, the human TR was compared with orthologous TRs in all outgroup species outside the clade. For example, 1,669 human TRs in our data set are perfectly conserved compared with one or more TRs in orthologs from any of the 21 nonmammalian species, providing evidence that these human TRs have been conserved at least since the root of all mammals (blue continuous curve). From more general to more specific clades, the number of human TRs with evidence for conservation at least to the root of the clade is cumulatively increasing. (*B*) The *y*-axis shows the number of human TRs separated compared with at least one other species within the clade. Denoted in red are the three measures of TR separation, where darker color marks the higher degree of separation. For example, 146 human TRs in our data set are perfectly separated compared with one or more TRs in orthologs from any of the other 39 mammalian species (red continuous curve). As the number of species in the clade wise comparison increases from Hominines to broader clades, the number of separated TRs is growing cumulatively.

Table 1 shows the frequency of orthologous TRs and their level of conservation for prominent species and the most frequent TR types. To investigate the significance of a given TR for the TR-containing gene, we first tested whether the TR was equally old as the TR-containing gene. For each human TR-containing gene, we traced back the most distant ortholog in the other species (fig. 3*A*). Similarly, for each human TR-containing gene, we traced back the most distant ortholog that still contained at least four TR units (table 1 and fig. 3*A*). We found that almost all TRs were as ancient as the TR-containing gene, and were lost only in rare cases. For example, of all human TR-containing proteins, 90% had an ortholog in a nonmammal species, of which 94% also contained the homologous TR with at least four units. This implies that the TR often is an essential component of the TR-containing protein.

### The Conservation of Human Protein TRs

To determine evolutionary ranges with evidence for TR conservation, we identified the species furthest to human for which either perfect conservation, or “strong conservation” was detected in the orthologous TR. For example, to establish conservation of a human TR at least to the root of the bilaterians, the human TR was compared with its orthologous TR—if present—in the nonbilaterian species available in our data set, namely *S. cerevisiae.* The TR conservation between human and *S. cerevisiae* provided strong evidence for the conservation of the human TR since their speciation, thus beyond the first bilaterians. Further, the conservation of a human TR compared with its ortholog in any of the other 60 species indicates that it has been conserved at least since the speciation of chimp and human, thus well beyond the most recent common ancestor of all humans. This definition is cumulative, that is, the number of TRs that are conserved to the root of a given clade (e.g., bilaterians) is less than (equal to) the number of TRs conserved to the root of any nested clade (e.g., chordates).

Figure 3*A* summarizes the numbers of human TRs that have been conserved since different eukaryotic speciation events, ordered from the most recent (human/chimp) to the oldest (human/yeast). We found that 92% of all human protein TRs was strongly conserved, and 90% was perfectly conserved between human and at least one other species in our data set. Surprisingly, such conservation was observed not only within closely related species: 61% of all protein human TRs were strongly conserved since the root of the mammalian clade, while 17% were strongly conserved since the root of the vertebrates. This shows that for the vast majority of TRs their mode of evolution is not marked by high rates of TR unit gain or loss.

### Functional Analysis of Strongly Conserved TRs

To better understand the functional constraints that require conservation of TRs, we contrasted the subset of human proteins containing TRs that were strongly conserved in at least one species beyond the mammals (1,896 TRs in 1,553 proteins) with all human TR-containing proteins (3,091 TRs in 2,532 proteins). We studied the distribution of different TR types, as well as the enrichment of functional annotations for proteins with TRs using GOrilla (Eden et al. 2009).

TR types strongly conserved at least to the root of all mammals are diverse, spanning 81% of all annotated human TR types. The distribution of different TR types among the proteins with conserved TRs is highly biased. For example, more than half (58%) of all conserved TRs could be attributed to just 5% of all TR types. In other words, a handful of TR types were observed at very high frequencies (e.g., zinc finger, ankyrins or ANKs, LRR, WD40), whereas the majority of others appeared at low frequencies.

Interestingly, for a range of TR types such as the Armadillo repeat (ARM), the HEAT repeat and the PHD-finger, all human TRs have been conserved since the ancestor of mammals. Likely, the unit configuration of these TR types is essential to maintain protein function, be it for structural reasons, or due to the function of certain amino acids on specific TR units.

**Fig. 4.**
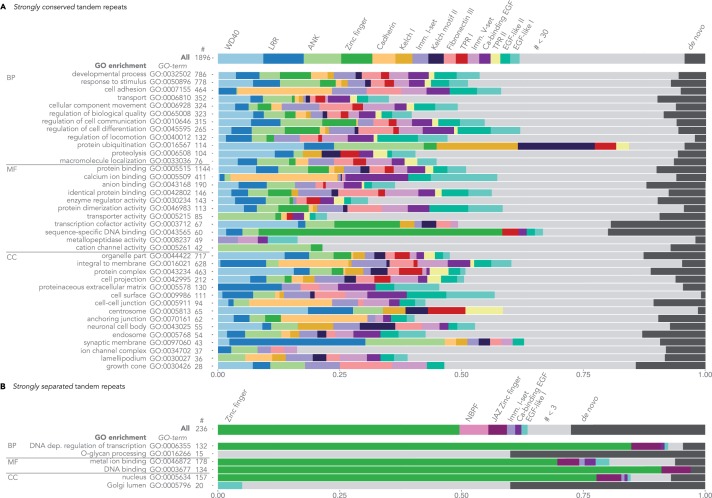
TR types and GO enrichment of human proteins with conserved and separated TRs. (*A*) TRs that have been strongly conserved at least since the root of mammals; (*B*) TRs that show strong separation compared with an orthologous TR in at least one other mammal. The first summary bar in each plot shows the frequency of the different TR types: there are 1,896 conserved TRs, with the WD40 TR being the most frequent, and 236 separated TRs, with the Zinc finger TR being the most frequent. All TR types based on de novo TR detections were binned into one category (denoted with dark gray), although they may describe very diverse motifs. Likewise, TR types based on PFAM annotations with low frequencies (<30 TRs for the set of strongly conserved TRs, and <3 TRs for the set of strongly separated TRs) were binned together (denoted with light gray). The thinner bars below the summary bars show representative enriched GO terms ordered by their frequency. Each bar corresponding to a GO term depicts the distribution of different TR types in proteins annotated with this GO term. GO terms are grouped by their respective ontology: Biological Process (BP), Molecular Function (MF), or Cellular Component (CC).

The high diversity of TR types found for conserved TRs was reflected in the diversity of functions performed by proteins with conserved TR types. A GO-term analysis of these proteins revealed enrichment in diverse biological processes, including prominently, stimuli response, cell adhesion, protein ubiquitination, locomotion, and regulation of development, particularly nervous system development. In terms of molecular function, proteins with conserved TR are enriched in particular in protein binding and catalytic activities (fig. 4*A*). To reveal the correlation of TR type and protein function, we linked the results of the GO-enrichment analysis to the TR types found in the protein with enriched functions, as summarized in figure 4*A*.

The TR type with the largest number of conserved TRs was WD40 (table 2). WD40 repeats are thought to form a β-propeller structure that serves as a rigid scaffold to mediate the assembly of multi-protein or protein–DNA complexes (Stirnimann et al. 2010; Xu and Min 2011). WD40 repeats interact with a wide variety of proteins, peptides and DNA, and are involved in diverse cellular functions facilitated by the large sequence diversity in the TR region (Stirnimann et al. 2010). At the same time, no WD40 repeat has been annotated with enzymatic function (Stirnimann et al. 2010). Accordingly, 50% (88/177) of all human WD40 repeat containing proteins with conserved WD40 repeats are involved in protein binding (GO:0005515).

Next to WD40 repeats, TRs with 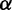-helical TR units of 20–40 aa including large groups such as LRRs, ANKs, ARMs, HEAT, and tetratricopeptide repeats are thought to be involved in protein binding (Groves and Barford 1999). The suprahelical structure of the TR region of such TRs is thought to form the scaffold for the assembly of multiprotein complexes (Groves and Barford 1999; Barford 2012; Javadi and Itzhaki 2013), which in return mediates protein binding. Gains/losses of TR units in the TR region are likely to cause changes in the scaffold structure of TRs mediating molecular interactions. Any such changes would consequently affect the interaction properties. These observations were consistent with our GO-term analysis showing that conserved TRs in particular were significantly enriched with protein binding (60%, fig. 4*A*).

Further, more than a third of all conserved TRs were part of membrane proteins (40%, fig. 4*A*). Among the most common TR types in this group were cadherins, ANKs, LRRs, and WD40 repeats (fig. 4*A*). Structurally, these TRs are mostly located in the extracellular matrix, or the cytosol, but not within the membrane (Angst et al. 2001; Hulpiau et al. 2013). Functionally, these TRs may be involved in protein binding outside the membrane (de Wit et al. 2011; Hulpiau et al. 2013; Mou et al. 2013). The most frequent conserved transmembrane TRs are transmembrane helices (TMH; PF00520) in ion channels.

### Human Proteins with Evidence for TR Separation

To determine evolutionary ranges with evidence for TR unit gains/losses, we identified the species closest to human for which either a difference in TR unit number, strong or perfect separation was detected in the orthologous TR. For example, a difference in TR unit number in orthologs of human and chimp would suggest that TR unit changes occurred in at least one of the two lineages since speciation, indicating that the TR is mutable on the time scale of the speciation of the hominines. Perfect separation indicates that gains or losses of units had occurred repeatedly, affecting all TR units within the TRs.

For different eukaryotic clades, we calculated the number of TRs that have undergone unit changes in at least one species within this clade (summarized in fig. 3*B*). Using this approach, for each TR we assessed when it was subjected to different degrees of unit gain/loss. Consequently we found that 5% (or 8%) of all human TRs were completely (or strongly) separated from at least one orthologous mammalian TR. In contrast, 61% of all human TRs were strongly conserved at least since the root of mammals. Note that it is possible that a TR was conserved in the human lineage beyond the ancestor of all mammals, while at the same time strong gains/losses leading to strong separation occurred on the lineage of another mammal after the separation from the human lineage. However, this is expected to be rare (0.5% of human TRs in our data), showing that within a given clade TRs consistently evolve by one single evolutionary mode, but not in a mixed-mode fashion.

Of particular interest is evidence for TR unit gains/losses in orthologous TRs within hominines, which would indicate that TR unit gains/losses might also occur on even shorter time scales, perhaps even on the population scale. However in our data, no human TR showed perfect separation compared with the orthologous TR in chimp or gorilla. In this range, only four TRs showed strong separation, including the TAPE repeat in a tumor necrosis factor (O14798, ENSP00000349324), and a de novo TR in the NAC-alpha domain-containing protein 1 (O15069, ENSP00000420477). In both examples, the TR consisted of almost identical TR units.

One other possibility is that TR unit gains/losses do occur, but do not affect the entire TR region. To account for this case, we also annotated pairs of TRs that exhibited a difference in TR unit number: 235 or 8% of all human TRs showed a difference in TR unit number compared with their orthologous TRs in chimp or gorilla, corresponding to 34% of all human TRs that had not been strongly conserved in the same range. In comparison, only 4 or 0.001% of all human TRs showed strong separation compared with their orthologous TR in chimp or gorilla. Thus, in nonconserved TRs, unit mutations often lead to a change in the number of TR units without affecting the entire TR region.

### Functional Analysis of Strongly Separated TRs

To shed light on TR characteristics that correlate with strong unit gains/losses, we contrasted the subset of proteins with perfectly or strongly separated TRs in at least one species within the mammals (236 TRs in 230 proteins) with all human TR-containing proteins (3,091 TRs in 2,532 proteins). Similarly to strongly conserved TRs, we analyzed separated TRs with respect to the distribution of TR types and the GO-term enrichment (fig. 4*B*).

More than half of all strongly separated TRs were formed by zinc finger motifs coordinating one eponymous zinc ion (table 1, supplementary fig. S6, Supplementary Material online). The family of zinc finger genes has been subject to a massive expansion in vertebrates, accounting for ∼2% of all human genes (Tadepally et al. 2008). The sheer number of zinc finger proteins suggests that correct orthology annotation might be particularly difficult in this group, potentially leading to an overestimation of the number of not-conserved TRs. The majority of the zinc-finger proteins bind to DNA, acting as highly specific transcription factors. More recently, binding to RNA and protein structures has also been observed (Gamsjaeger et al. 2007). Taken together, proteins with zinc finger TRs can explain most of the enriched GO terms for strongly separated TRs (fig. 4*B*). Most of the zinc-finger-containing genes are arranged in clusters, and evolve through tandem gene duplications and losses (Tadepally et al. 2008). Thus, possibly, the same evolutionary gain/loss mechanisms promote the evolution of the zinc finger TRs. Moreover, we noted that single zinc finger TR units often occupied exactly one exon. Although such zinc fingers clearly are repeated in tandem within the protein sequence, the TR units appear disconnected on the DNA sequence.

Among the other TRs strongly separated in at least one mammal was the neuroblastoma breakpoint family (NBPF or DUF1220, PF06758), immunoglobulin I-set (PF07679), the calcium-binding EGF (PF07645), and an EGF-like (PF00008) domain repeats. In total, 52 distinct TR types were subject to strong separation in at least one human protein, with 30 of these being de novo annotations. The abundance of de novo detected TRs might imply that the TR types that undergo strong gains/losses in many cases may be relatively rare types, which have possibly appeared recently.

## Discussion

In our proteome-wide analyses, most of the TRs were remarkably informative about their duplication history, despite their short sequence. As a result, we were able to classify the majority of human TRs as conserved (68%) with well-preserved TR unit configurations over long evolutionary distances (at least to the root of all mammals), while only few TRs were separated (8%) with clear evidence of configuration changes in the same range. Below we discuss these sets of TRs, as well as the correlation of their evolutionary mode with TR characteristics including the TR unit number, length, between-unit divergence, as well as the exon structure underlying the TR region.

### Rapid Evolution of Protein Tandem Repeats Is Rare

Very few TRs appear to undergo rapid TR unit gains/losses. However, these few identified examples of separated TRs might exhibit variation within populations. Indeed, they include the zinc finger repeat in PRDM9, which carries strong variation in both chimp and human populations (Hinch et al. 2011; Auton et al. 2012), and strongly influences the location of meiotic recombination hotspots (Baudat et al. 2010; Berg et al. 2011). Further, 12 (of 14) NBPF repeats involved in higher cognitive functions show a difference in TR unit number between human and chimp and gorilla. The majority of NBPF repeats were not strongly conserved between human and any other species, with none of them were strongly conserved beyond the Catarrhines. Similar to zinc fingers, frequent NBPF unit gains/losses coincide with the recent expansion of TR-containing genes, particularly in the human lineage (Popesco et al. 2006) where gene copy number variation correlates to neurodevelopmental disorders (Dumas et al. 2012) and brain cancer (Diskin et al. 2009).

Beyond these (few) examples our analysis shows that separation of TR units is extremely rare. Strikingly, no case of perfect separation was found between human and chimp or gorilla. In comparison, other types of sequence changes are much more common in this range: ∼70% of all proteins were subject to substitutions (median of two nonsynonymous substitutions per protein), and ∼5% were subject to in-frame indels in a comparison of the human and chimp proteomes (Chimpanzee Sequencing and Analysis Consortium 2005). In summary, the vast majority of analyzed TRs cannot present potential for population level variability in terms of tandem repeat unit gains/losses, and so this process is unlikely to facilitate rapid adaptation to changes as has been proposed (e.g., Chevanne et al. [2010] for a WD40 TR in *Podospora anserina*).

Interestingly, more TRs with difference in TR unit number were annotated de novo compared with all TRs (e.g., within hominines this was 22% vs. 12%, respectively). Having fewer Pfam annotations among variable TRs might indicate that de novo TRs are rare (otherwise they should be represented in Pfam), and therefore more likely recent. Indeed, 75% of those variable de novo TRs had no ortholog TR outside mammals, compared with 15% in the complete TR set. Our findings are consistent with a recent hypothesis that TRs may function as a substrate for the formation of new domains and subsequently new genes (Bornberg-Bauer and Albà 2013).

### The Majority of Human Protein TRs Are Highly Conserved

The majority of all human TRs are conserved at least within mammals or further back in time—in stark contrast with the rarity of separated TRs. Most likely, these conserved TRs had avoided any recent TR unit changes, with a unit configuration conserved deep into the eukaryotic tree. Thus, the TR duplications that had lead to the original expansion of the TR should be ancient. Indeed, 52 TRs were conserved even between human and yeast, amounting to 13% of all human TRs that have a detectable ortholog in yeast. These ancient, highly conserved TRs include a range of TR types: for example, domain repeats such as the calponin homology (PF00307) and the prenyltransferase (PF00432), solenoid repeats such as WD40 (PF00400) (fig. 1*B*), armadillo (PF00514), and also repeats with other structural configurations such as the EF hand (PF00036) (see Kajava 2012 for a structural classification of TRs). With such structural diversity at hand, there must be more than one guiding principle to explain the structural importance of ancient conserved TRs.

To investigate whether the conservation of a TR unit configuration is generally accompanied by the conservation of the TR unit sequence, we estimated the relative substitution rates in the TR and flanking regions (see Materials and Methods). We found that the substitution rates in the TR region of strongly conserved repeats were on average 2.3 times lower than the rates in the protein sequence flanking the TR, both of which were by an order of magnitude below the respective rates for strongly separated TRs (supplementary table S1, Supplementary Material online). This shows that the majority of TRs are conserved both in terms of the TR sequence and in terms of their unit configuration (allowing accurate reconstruction of TR unit phylogenies over long evolutionary timescales). Such sequence conservation on two levels is likely to be accompanied by an equally sustained structural conservation of TR regions, which is presumably required to maintain the function of the TR-containing protein.

### TR Conservation and TR Type

Many more human TRs are conserved rather than separated (e.g., ∼8:1 in mammals). Conserved TRs clearly encompass more distinct TR types compared to separated TRs (∼3:1), although the ratio is lower due to the relatively large number of de novo TRs among the separated repeats. Interestingly, TRs of the same type may be found in proteins with either conserved or separated TRs (such as zinc finger, Ca-binding EGF and the Immunoglobin I-set domains in fig. 4). For example, among the zinc finger TRs 121 were strongly conserved to the root of all mammals, while 117 were strongly separated in the same range. Generally however, different TR types dominated the sets of conserved and separated TRs, with a clearly larger variety among the conserved TRs (fig. 4; supplementary figs. S5 and S6, Supplementary Material online).

Similarly, the proteins containing either conserved or separated TRs differed in their functional annotations. Proteins with conserved TRs were enriched not only in a vast variety of molecular functions, related for example to cell–cell communication and cell adhesion, regulation of (nervous system) development, protein binding, but also catalytic activity. On the other hand, functions of separated TRs were dominated by DNA binding and gene expression regulation (largely due to zinc finger TRs).

### TR Conservation and Substitution Rates

**Fig. 5.**
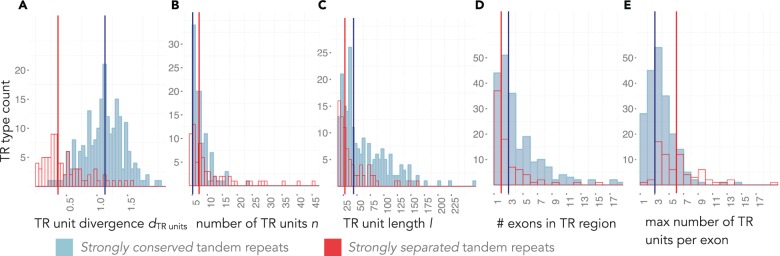
Characteristics of separated versus conserved tandem repeats. Shown are frequency distributions of TR characteristics (see Materials and Methods) for strongly conserved (blue) and strongly separated (red) human TRs, with the mammalian clade as the reference. For each TR type defined by distinct circular HMMs, the mean value was calculated for each characteristic. For example, the mean number of zinc finger TR units was 7 for conserved TRs and 13 for separated TRs, each constituting one data point summarizing a large family of zinc fingers. The total data set comprises average values for 235 TR types with strongly conserved TRs and 86 TR types with strongly separated TRs.

In our data set, the mode of evolution of a given TR was best predicted by its between-unit sequence divergence (fig. 5*A*). TR units in strongly separated TRs clearly had a lower divergence compared with TR units in strongly conserved TRs. At the same time, we found that substitution rates in the TR regions of strongly separated repeats were on average ten times higher than those for strongly conserved TRs (in a comparison of human/mouse TR containing orthologs (details in Materials and Methods; results in supplementary table S1, Supplementary Material online). Taken together, strongly separated TRs had lower between-unit sequence divergence, despite higher substitution rates in the whole TR regions. The following may explain these apparently contradictory results. In strongly separated TRs, due to the elevated rates of TR unit gain/loss, some TR units repeatedly get lost, while others duplicate in identical copies. As long as the substitution rates do not exceed the unit gain/loss rates, the TR units within the TR region will preserve high similarity (or low divergence) with each other (Chevanne et al. 2010). Despite the similarity of TR units between each other, the effective substitution rate still appears high when comparing orthologous TR regions: When substitutions occur on the propagating TR unit in one ortholog, these will spread over the entire TR region, leading to the divergence of the orthologous TR regions. Moreover, the accumulation of substitutions/indels in the repeat unit sequence is thought to decrease the rate of TR unit gain/loss by lowering the sequence mispairing probability (Schug et al. 1998; Faux et al. 2007). Therefore, low TR unit divergence within a TR region may be likewise a consequence and a requirement for TR unit gains/losses. Speaking now of conserved TRs, their high dissimilarity level is explained by their ancient origins that outweigh their low evolutionary rates.

### TR Conservation and the Number of TR Units

Separated TRs tended to contain more TR units than conserved TRs (fig. 5*B*). The tendency of separated TRs to contain more units may be grounded in molecular biases: For nucleic tandem repeats, it has been observed that TRs with more units are subject to increased duplication rates (Schlötterer 2000; Ellegren 2004; Bhargava and Fuentes 2010), presumably due to a larger number of potential slippage sites. Similarly, protein TRs with a higher number of units may be more likely to undergo TR unit number changes.

In general, it is interesting to understand why for the majority of TRs the number of TR units is constant throughout large evolutionary time scales. Most likely, the protein function, and in turn its structure necessitate a fixed number of TR units. Interestingly, conserved TRs of the same type may appear in different (nonorthologous) proteins with widely varying numbers of TR units. For example, in conserved human zinc finger TRs unit numbers ranged from (the minimum of) 4 to 39. This holds for TRs that fold into the “beads on a string” structure such as the zinc finger TRs (Kajava 2012), but also for TRs where the individual TR units do not fold independently, such as the EGF-like laminins (up to 31 units), the linear/open solenoid LRRs (up to 27 units), and the circular/closed solenoid WD40 repeats (up to 37 units). WD40 repeats were also shown to be highly mutable in one fungi gene family and still functional for different numbers of TR units (Chevanne et al. 2010). All in all, for many TR types, a fixed number of TR units is not per se crucial to guarantee the functioning of the TR-containing proteins, in agreement with results by other authors (e.g., Abraham et al. 2009). There must be additional reasons to explain the high conservation of the majority of TRs, such as the necessity to provide a defined scaffold structure to mediate protein binding (see Results).

### TR Conservation and the TR Unit Length

Separated TRs exhibited shorter repeat units compared with conserved TRs (fig. 5*C*). For nucleic microsatellite and minisatellite TRs, for example, TRs with shorter units also have higher TR unit duplication rate (Schlötterer 2000; Leclercq et al. 2010). If this were applied to protein TRs, those with shorter TR units would be expected to undergo more TR unit changes and are thus more likely to become separated.

In light of the earlier discussion, it does not seem surprising that for minisatellite type TRs with shorter units (10–15 aa) we observed a clear decrease in unit conservation, as well as an increase in the relative proportion of strongly separated TRs, compared with TR regions with longer units (supplementary fig. S4, Supplementary Material online). Possibly, as the TR unit length decreases there would be a transition from observing mostly conserved TRs to gradually observing more and more separated TRs. Indeed, frequent insertions/deletions have been observed for homorepeats and dipeptide repeats among primates, including human (Loire et al. 2013). In particular, variation in the number of TR units in protein homorepeats has been reported within human populations, and is known to be associated with various human diseases (Orr and Zoghbi 2007). On the other hand, human homorepeats were shown to be more conserved than corresponding trinucleotide TRs in noncoding sequence (Mularoni et al. 2010). Note that with shorter TR units, phylogeny reconstruction becomes increasingly prone to errors, which may obscure TR phylogeny-driven results for such ranges.

### TR Conservation and the Exon Structure

Frequently, exons in TR-containing proteins span multiples of whole TR units (Street et al. 2006; Björklund et al. 2010). TRs may evolve by a mechanism of tandem gains/losses of repeat units such as replication slippage, or alternatively by duplication of whole exons, which does not necessarily occur in tandem, such as exon shuffling (Björklund et al. 2006). To distinguish between these mechanisms, we measured the number of exons spanned by the TR region, as well as the maximum number of adjacent TR units found in a single exon (fig. 5*D* and *E*).

In our data, 31% of all separated TRs were contained within a single exon. For these, we can exclude the exon-shuffling-like process to explain TR unit changes. On the other hand, for 26% of all separated TRs, one exon corresponded to at most one TR unit. Lacking proximity of the protein TR units in the nucleic sequence, these TRs most likely evolve through an exon shuffling-like process. Another indicator for the mechanism of TR units gains/losses can be derived from the exon structures of multiple TRs of the same TR type. For example, we found that whilst the number of NBPF TR units varied widely (up to 55) in all 14 NBPF-containing proteins, the number of TR units per exon stayed constant (2 or 3). This indicates that NBPF TRs evolve through an exon-shuffling-like process.

Altogether, both mechanisms of unit gains/losses seem to play an important role during the TR evolution. Interestingly, many of the separated TRs either were found to occupy one exon per TR unit, or contained many TR units per exon, exhibiting a roughly bimodal distribution in terms of the maximum number of TR units per exon (fig. 5*E*), whereas the conserved TRs did not show this behavior. Possibly, for some conserved TRs the presence of multiple exon boundaries rupturing the TR unit structure on the nucleic level may prevent duplications/losses of TR units.

## Conclusion

Our genome-wide study of the evolution of human protein TRs demonstrates that despite the common belief that TRs evolve rapidly, large numbers of protein TRs (*l* ≥ 15 aa) exhibit sustained conservation deep into the eukaryotic tree, with many TR regions preserved even since the common ancestor of human and yeast. Surprisingly, TR regions are frequently the most conserved part of the protein sequence. Conserved TRs can be found in proteins performing a wide variety of key functions. All together, our observations suggest a pronounced role of protein TRs in the function of the TR-containing protein, indicating that the functional significance of TRs has been underestimated. On the other hand, we found only few TRs with evidence for recent and strong TR unit gains/losses. To better understand the functional and potentially adaptive relevance of this small set of fast evolving protein TRs in the future, a casewise analysis of their function may be of interest.

Cross-species studies of tandem repeat unit phylogenies, like the one presented here, appear to be a powerful tool to gain insights on TR evolution. We found that human TR sequences are for the majority of TRs remarkably informative about their duplication history. This opens the door to more detailed studies of TR unit gains/losses. Possibly, unique events can be pinpointed to specific lineages within the gene phylogeny, but also within the TR region. Future research on the association of specific 3D structures and functions to TRs in proteins could use the analyses of TR unit phylogenies to provide insights to the impact of specific TR unit gains/losses.

## Materials and Methods

### Annotation of TRs in Human Proteins with Circular HMM

The complete set of 20,240 gene trees with associated protein sequences from 61 eukaryotic species including human were obtained from Ensembl Compara v69. For all human sequences, TRs were annotated based on: 1) tandemly repeated PFAM A domains and 2) de novo detections.

For each PFAM A domain annotated in the Ensembl human proteins, the corresponding sequence profile HMM was obtained from the PFAM database (Punta et al. 2011). To detect PFAM domains that occurred as TRs, their profile HMMs were transformed into circular profile HMMs, or cpHMMs (fig. 2), so that one motif (described by its sequence profile) could be repeated in tandem via a circular transition from the final to the starting state of the HMM. TRs corresponding to PFAM A domains were annotated in human sequences using the Viterbi algorithm applied to cpHMMs (supplementary fig. S1, Supplementary Material online). Annotated this way, TRs were retained for further analyses if they had at least four TR units (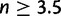).

To include TRs that were not represented among PFAM A, additional TRs were predicted de novo on the human proteome with HHrepID v1.1.0 (Biegert and Söding 2008), T-REKS v1.3 (Jorda and Kajava 2009), TRUST v1.0 (Szklarczyk and Heringa 2004) and XSTREAM v1.72 (Newman and Cooper 2007) and subsequently filtered for minimal requirements (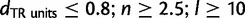) (for exact definitions of 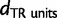, *l* and *n,* see the later discussion) and statistical significance (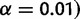. Statistical significance of TR predictions was assessed using the likelihood ratio tests as in (Schaper et al. 2012), for details see supplementary figure S1, Supplementary Material online. De novo predicted TRs overlapping with PFAM-based TR annotations were discarded. Where de novo TRs overlapped, only the best prediction (with the highest statistical significance and the lowest TR unit divergence) was used for further analyses. Profile HMMs of de novo TRs were built using HMMER (Eddy 1998), and are available at http://www.atgc-montpellier.fr/TRE (last accessed February 20, 2014). Again, we refined the de novo based TR annotation of human proteins using cpHMM, and statistically validated all refined TRs (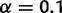) retaining those with at least four TR units (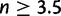) and unit length 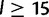. Note that due to the Markovian property of the cpHMM, the annotated TRs will not be biased to a particular number of TR units.

### Phylogenetic Analysis of TRs within the Eukaryotic Clade

For every human TR, we used its cpHMM to annotate homologous TRs in all orthologous (including 1:1, 1:many, and many:many orthologs) genes from other eukaryotes as represented by the Ensembl Compara gene trees. Next, we built MSAs (Multiple Sequence Alignments) for each ortholog pair (supplementary fig. S1, Supplementary Material online). For each TR MSA that contained at least four TR units in both orthologs, we reconstructed bispecies maximum likelihood TR unit phylogenies using PhyML 3.0 (Guindon and Gascuel 2003; Guindon et al. 2010) with default options (LG+Γ model; examples in fig. 1).

### TR Characteristics

We correlated TR classification with a range of TR characteristics (fig. 5). For this purpose, we considered TRs that were classified as strongly conserved since the root of all mammals, or as strongly separated between human and at least one other mammal. For each single TR, we calculated the following characteristics:

- TR unit length 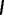, defined as the number of noninsertion sites of the TR unit, parsimoniously assuming an insertion if at this site (in the respective column of the TR MSA) the observed amino acid characters are at least as many as gaps.
- (Effective) number of TR units 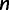, as the total number of noninsertion amino acid sites in the TR-MSA divided by 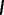.
- TR unit divergence 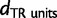, maximum likelihood estimate of the TR unit divergence obtained as a by-product of the model-based TR significance test (Schaper et al. 2012): 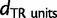 is measured in expected number of aa substitutions per site since the most recent common TR unit ancestor.
- The number of exons spanning the TR region at least partly.
- The maximum number of complete TR units in a single exon. The last two statistics relied on the exon structure of the human TR-containing proteins according to Ensembl v.69.

### Function Enrichment Analysis

Ensembl protein identifiers were mapped to HGNC symbols. The mapping of HGNC symbols to GO functional annotations, and the enrichment analysis assuming a hypergeometrical model was conducted with Gorilla (Eden et al. 2009). All TR-containing human proteins constituted the background distribution, which was independently contrasted with distributions of functions within strongly separated and strongly conserved sets of TRs. The complete enrichment data set including directed acyclic graphs of enriched GO terms is available at http://www.atgc-montpellier.fr/TRE (last accessed February 20, 2014).

### Substitution Rates in TR Regions and anking Protein Sequence

For all pairwise alignments of human–mouse TR-containing orthologs in Ensembl Compara, the evolutionary distances between the TR regions in both species (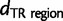), and the corresponding flanking protein regions in both species (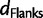) were computed separately (using LG + Γ in PhyML 3.0). For this purpose, the flanks on either side of the TR region were concatenated. The computed evolutionary distances are equivalent to estimates of substitution rates per site. The boundaries of TR region and flanking region were taken as the mean of the predicted TR boundaries in both species (if different).

### Statistical Significance of Assigning TRs as Conserved Based on TR Unit Phylogenies

The probability of falsely assigning perfect conservation to a pair of random TRs with *n* units is as low as 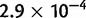 (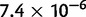) for *n* = 4 (5), rendering an overestimation of TR conservation unlikely (see the derivation discussed later). In comparison, inference errors in orthology annotation and phylogeny reconstruction are disproportionally more likely to obscure a perfectly conserved TR. Thus, the observed number of conserved TRs is presumably a lower boundary to the actual number of conserved TRs.

### Derivation of the Probability of Randomly Drawing Conserved TR Unit Phylogenies

**Formula**

Let 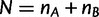 be the total number of leaves in an unrooted binary tree, with 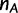 leaves representing TR units from species *A*, and 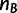 leaves representing TR units from species *B*. Assume 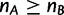 without loss of generality. For 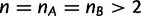, the probability of drawing a random phylogeny with perfectly conserved TR units under the uniform tree model is
(1)
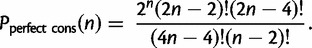

**Proof**

In a phylogeny with perfectly conserved TR units a) all leaves are paired in cherries and b) each cherry groups TR units one from each of *A* and *B* so that Kendall’s 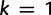 (i.e., the 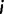
th TR unit in *A* is always paired with the 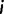
th TR unit in *B*).

a) The probability distribution of 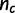 cherries in a random phylogeny with 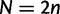 leaves drawn from the uniform tree model is (Hendy and Penny 1982; McKenzie and Steel 2000)

(2)
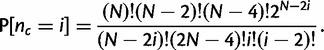


Thus, the probability that all leaves are paired in cherries (so the number of cherries is exactly *n_c_* = *n*) is
(2′)
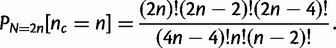

b) The probability of 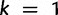 for a given topology with 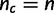 cherries is

(3)
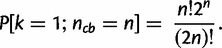



Here, we first used that the value of 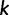 is independent of the order of cherries and assumed the cherries to be ordered. The probability is then given by the number of leaf assignments such that 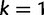, that is, 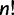, divided by the total number of distinct leaf assignments, that is 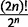. Finally,

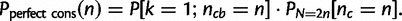



Table 2 shows these probabilities for a range of 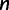.

**Formula**

For 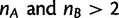 the probability of drawing a random phylogeny with strongly conserved TR units under the uniform tree model is
(3)
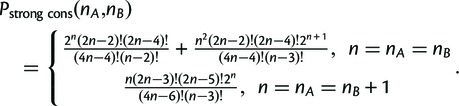

**Proof**

Strong conservation is assigned if 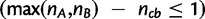 and Kendall’s 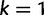. Thus, either a) the TR is perfectly conserved for 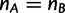, with probabilities derived earlier, or b) 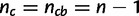 and 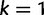 for 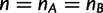, or c) 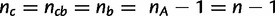 and 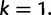 The probabilities for b) and c) can be derived by adapting (2) and (3):

b) Assumed is a topology with 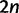 leaves so that there are 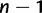 perfectly conserved cherries. The probability that the two leaves that are not part of a cherry hold TR units from both *A* and *B* is 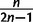, so that the total probability of case c) is

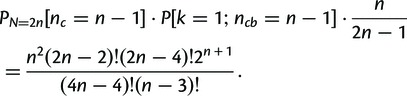

c) Assumed is a topology with 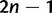 leaves and 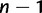 perfectly conserved cherries. Analogous to b), we consider that the probability that the one leaf that is not part of a cherry holds a TR unit from *A* is 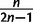, so that the total probability of case c) is

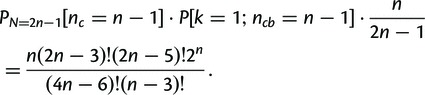



The formula follows.

### Statistical Significance of Assigning TRs as Separated from Bispecies TR Unit Phylogenies

The probability of falsely assigning perfect separation to a pair of random TRs with *n* TR units is 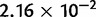 (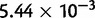) for *n* = 4 (5) (see the derivation later), which is elevated compared with the probability of falsely assigning perfect conservation. Inference errors in phylogeny reconstruction may still tend to cause an underestimation of the number of separated TRs. On the other hand, errors in sequencing and orthology annotation are expected to lead to an overestimation of the number of TRs that are separated or show a difference in TR unit number.

### Derivation of the Probability of Randomly Drawing Separated TR Unit Phylogenies

Any perfectly separated bisample TR unit phylogeny has exactly one bipartition separating the tree into two subtrees each of which with leaves representing TR units from only one species either *A* or *B* (see fig. 1*C* for one such configuration). The parsimony score of such a phylogeny is 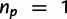.

**Formula**

Let 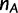 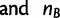 be numbers of TR units in species *A* and *B*, so that *N = *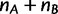 is the number of leaves in the unrooted binary tree of all TR units. The probability of drawing a random phylogeny with perfectly separated TR units under the uniform tree model is
(5)
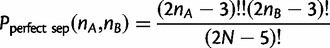

**Proof**

Since the number of distinct rooted binary trees with 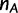 leaves is 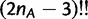 (Schröder 1870) the number of distinct perfectly separated trees connected at their roots is then 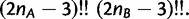 Given that the total number of distinct unrooted binary trees with 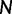 leaves is 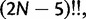 the probability of drawing a random tree with perfectly separated TR units from a uniform tree distribution is

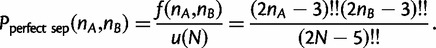

With the results of Carter et al. (1990) and Steel (1993) on the equivalent minimal coloring problem, it can additionally be shown that the probability of strong separation is
(6)
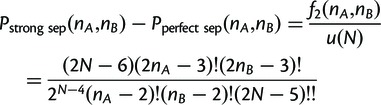



For equations (5) and (6) calculated probabilities for a range of 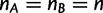 are shown in table 2.

## Supplementary Material

Supplementary figures S1–S6 and table S1 are available at *Molecular Biology and Evolution* online (http://www.mbe.oxfordjournals.org/).

Supplementary Data
