# Supplementary material for: Deep Conservation of Human Protein Tandem Repeats within the Eukaryotes
Source: Mol Biol Evol. 2014 Feb 3;31(5):1132–48. doi: 10.1093/molbev/msu062 (PMC3995336; doi:10.1093/molbev/msu062)
Supplement: Supplementary Data [file supp_31_5_1132__index.html]

Deep conservation of human protein tandem repeats within the eukaryotes — Deep Conservation of Human Protein Tandem Repeats within the Eukaryotes — Deep Conservation of Human Protein Tandem Repeats within the Eukaryotes — Supplementary Data 

# Deep Conservation of Human Protein Tandem Repeats within the Eukaryotes

## Supplementary Data

files

**Files in this Data Supplement:**

- Supplementary Data - pdf file
