# Supplementary material for: Deep Conservation of Human Protein Tandem Repeats within the Eukaryotes
Source: Mol Biol Evol. 2014 Feb 3;31(5):1132–48. doi: 10.1093/molbev/msu062 (PMC3995336; doi:10.1093/molbev/msu062)
Supplement: Supplementary Data [file supp_msu062_Supplementary_Material.pdf]

Supplementary Material to

Deep conservation of human protein tandem repeats within the eukaryotes.

Elke Schaper[1,2,3,4], Olivier Gascuel[4] and Maria Anisimova[1,3,4]

[1]Department of Computer Science, ETH Zürich, Zürich, Switzerland;
[2]Institute of Integrative Biology, ETH Zürich, Zürich, Switzerland;
[3]Swiss Institute of Bioinformatics (SIB), Lausanne, Switzerland;
[4]Institut de Biologie Computationnelle, LIRMM, CNRS – Université Montpellier 2, France.

S1 Further details of TR annotation

S1.1 Significance filtering of TRs

As we have shown previously, TR annotations need to be accompanied by rigorous statistical filtering to control the number of false positive predictions (Schaper et al., 2012). Therefore, in this study the statistical significance of a TR was determined by the likelihood ratio test comparing whether it is more likely that all repeat units stem from a common ancestor than being unrelated (Schaper et al., 2012). As part of the calculation, a maximum likelihood estimator of the TR unit divergence $d_{\text{TR units}}$ was derived.

For simplicity and due to limited TR lengths, we model TR phylogenies by an ultra-metric star tree (as in Schaper et al. (2012)), assuming that substitutions are described by the LG model (Le and Gascuel, 2008).

Indels were modelled by exponentially distributed waiting times between indel events (with indel mutation rate $\mu = 0.001$), and indel lengths were modelled by the Zipfian distribution (with parameter $a = 1.821$, following Chang and Benner (2004)). Gaps of the same length and same position within the TR MSA, as well as flanking gaps were ignored.

De novo TR detections were filtered at the significance level $\alpha = 0.01$ with number of TR units $n \geq 2.5$, unit length $l \geq 10$ and divergence $d_{\text{TR units}} \geq 0.8$, while human TR cpHMM annotations were filtered with $\alpha = 0.1$ and $n \geq 2.5$, and $l \geq 10$.

Overlapping of TR annotations was defined across all human paralogs related by the same Ensemble gene tree. If a *de novo* annotated TR overlapped with a PFAM annotated TR on a paralogous gene on the Ensembl MSA of both genes, the *de novo* annotated TR was discarded from the dataset.

S1.2 Annotation of protein tandem repeats with circular Hidden Markov models

To annotate TRs on a protein sequence with the circular HMM, the Viterbi algorithm was used. The result is the Viterbi path - the path through the circular HMM that best explains the protein sequence, which is interpreted as an emission instance of the model. The Viterbi path divides the protein sequence in the flanking sequence and the TR sequence. Next, all TR units were reconstructed, introducing TR unit breaks between the kth and the $k+1$th consensus position (cnf. Fig. 1). k was chosen so to minimise the distance between the break and the first consensus state, plus the distance between the break and the final consensus state. A TR MSA was calculated with MAFFT (v7.017b; default parameters) (Katoh and Toh, 2008).

For pairwise TR unit phylogeny reconstruction, an MSA of all TR units from both orthologous proteins was needed. Here, a single k was optimised for both TRs so to introduce the TR units breaks at the same consensus state. This might lead to incomplete first and last TR units (cnf. Fig. 1), which might disturb the TR unit phylogeny reconstruction. We discarded the flanking TR units in the pairwise MSAs in case they were shorter than $0.6l$.

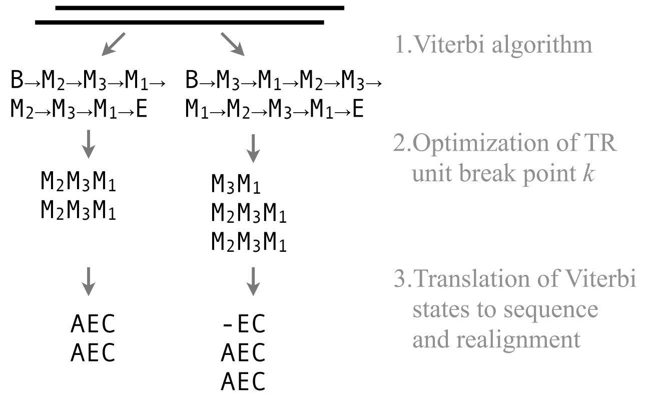

Suppl. Fig. S 1: From sequence to TR predictions using circular profile HMMs.

S2 Strongly conserved TRs

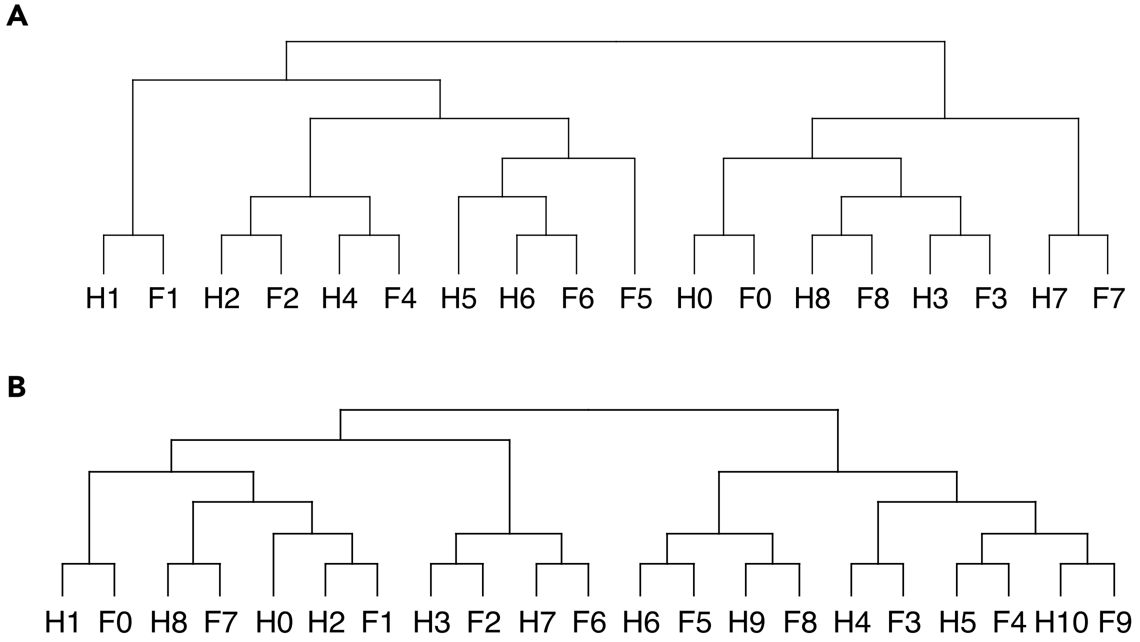

Suppl. Fig. S 2: **Examples of strong TR unit conservation.**
(A) Pairwise TR unit phylogeny of a leucine rich repeat (PF00560) in human (H; ENSP00000400803) and ferret (F; ENSMUP0000009083). For this phylogeny, it is $n_H = 9$, $n_F = 9$, $n_c = 8$, $n_{cb} = 8$, $k = 1$, $n_p = 9$.
(B) Pairwise TR unit phylogeny of a low-density lipoprotein receptor domain repeat (PF00057) in human (H; ENSP00000260197) and ferret (F; ENSMUP0000005716). For this phylogeny, it is $n_H = 11$, $n_F = 10$, $n_c = 10$, $n_{cb} = 10$, $k = 1$, $n_p = 10$.

S3 Strongly separated TRs

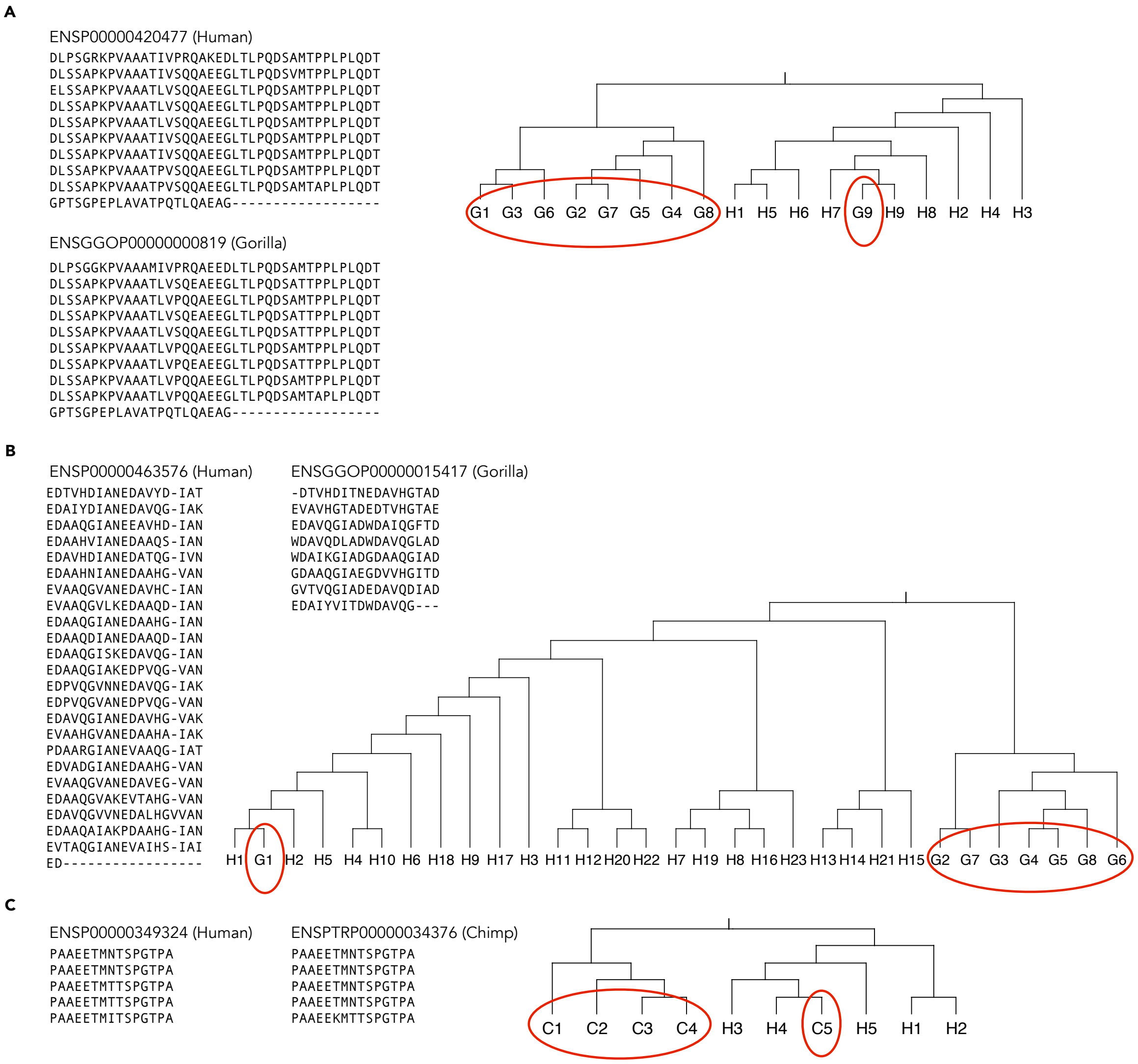

Suppl. Fig. S 3: **Examples of strong TR unit separation.** Pairwise TR unit phylogenies of *de novo* TRs. All clusters of non-human TR units are marked in red.
(A) human NAC-alpha domain-containing protein 1 (H; ENSP00000420477) and gorilla (G; ENSGGOP00000000819). It is $n_H = 9$, $n_G = 9$, $n_c = 4$, $n_{cb} = 1$, $n_p = 2$.
(B) human (H; ENSP00000463576) and gorilla (G; ENSGGOP00000015417). It is $n_H = 23$, $n_G = 8$, $n_c = 9$, $n_{cb} = 1$ $n_p = 2$.
(C) human tumor necrosis factor receptor superfamily member 10C (H; ENSP00000349324) and chimp (C; ENSPTRP00000034376) and associated TR unit MSAs. It is $n_H = 5$, $n_C = 5$, $n_c = 3$, $n_{cb} = 1$, $n_p = 2$.

S4 Evolutionary mode of human protein TRs for different TR unit lengths

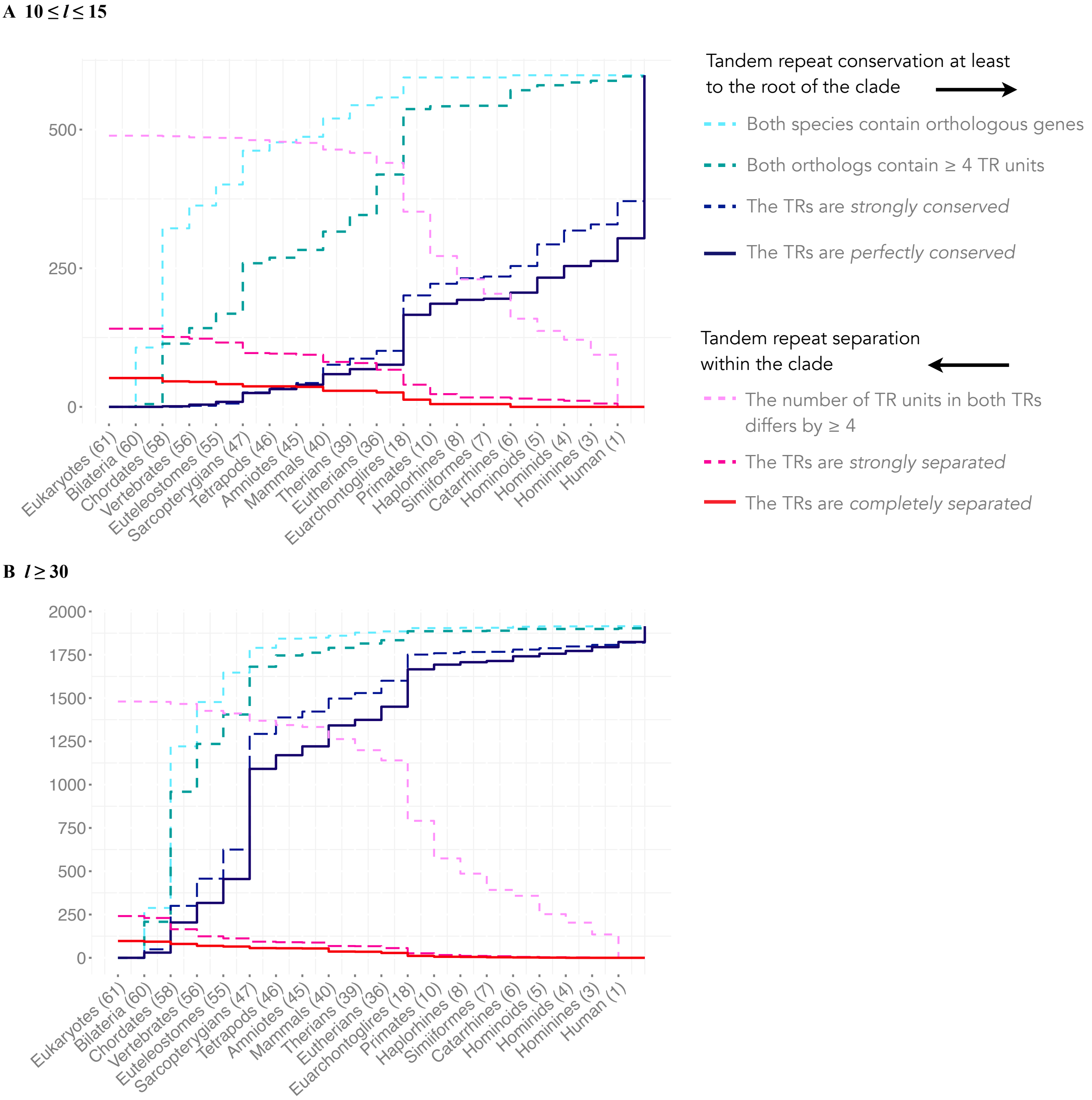

Suppl. Fig. S 4: **Conservation and separation of human protein TRs across the eukaryotes for different TR unit lengths.** **A** Results for 598 TRs with $10 \leq l < 20$. **B** Results for 1915 TRs with $l \geq 30$. For the full description of the shown results see the main manuscript, Fig. 3

S5 Evolutionary mode of human protein TRs averaged over TR types

A PFAM TR wise

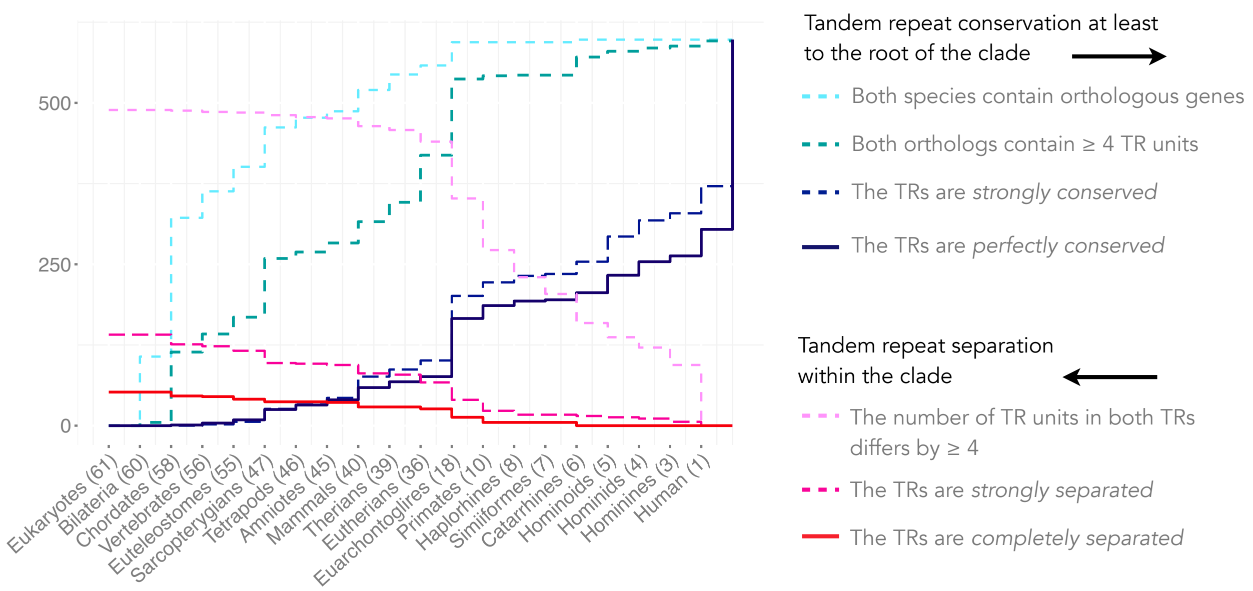

B *de novo*

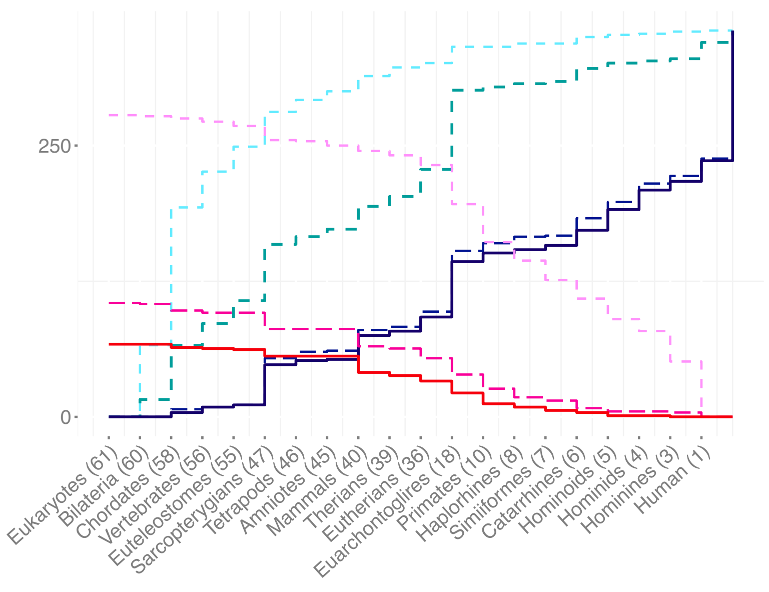

Suppl. Fig. S 5: **Conservation and separation of human protein TRs across the eukaryotes. A The average result is shown for all TRs of the same PFAM type.** For the full description of the shown results see the main manuscript, Fig. 3

S6 Evolutionary mode of human protein TRs for different PFAM types

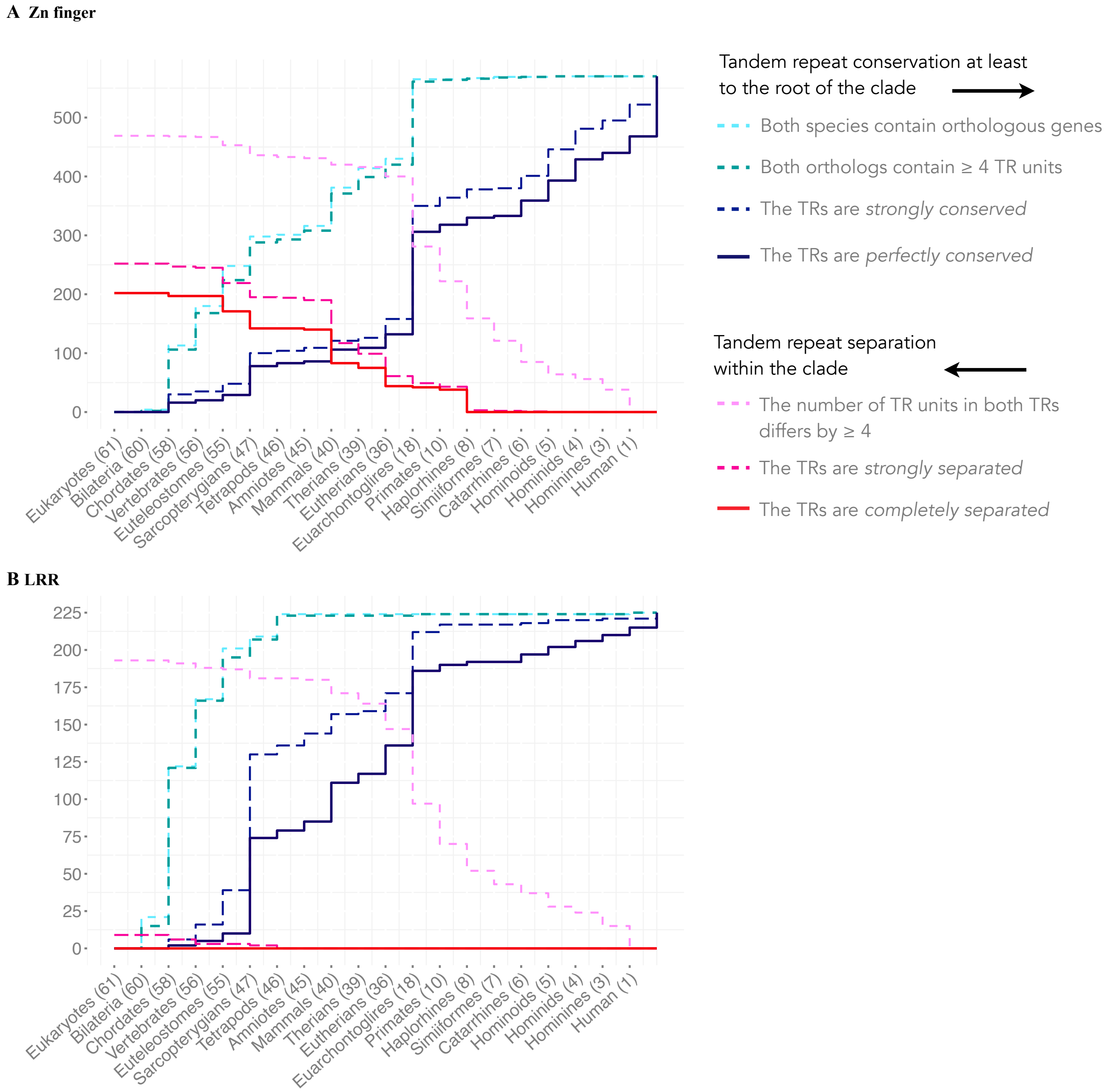

Suppl. Fig. S 6: **Conservation and separation of human protein TRs across the eukaryotes for different PFAM families.** For the full description of the shown results see the main manuscript, Fig. 3

C WD40

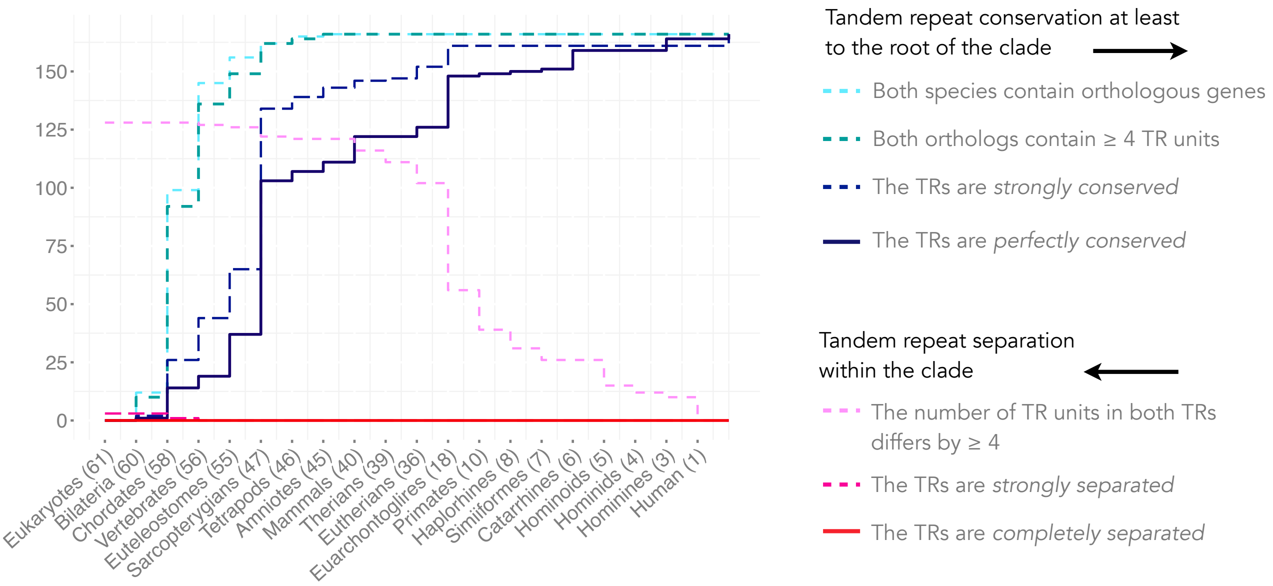
150
125
100
75
50
25
0
Eukaryotes (61)
Bilateria (60)
Chordates (58)
Vertebrates (56)
Euteleostomes (55)
Sarcopterygians (47)
Tetrapods (46)
Amniotes (45)
Mammals (40)
Therians (39)
Eutherians (36)
Euarchontoglires (18)
Primates (10)
Haplorhines (8)
Simiiformes (7)
Catarrhines (6)
Hominoids (5)
Hominids (4)
Homininae (3)
Human (1)
Tandem repeat conservation at least to the root of the clade
Both species contain orthologous genes
Both orthologs contain ≥ 4 TR units
The TRs are *strongly conserved*
The TRs are *perfectly conserved*
Tandem repeat separation within the clade
The number of TR units in both TRs differs by ≥ 4
The TRs are *strongly separated*
The TRs are *completely separated*

D ANK

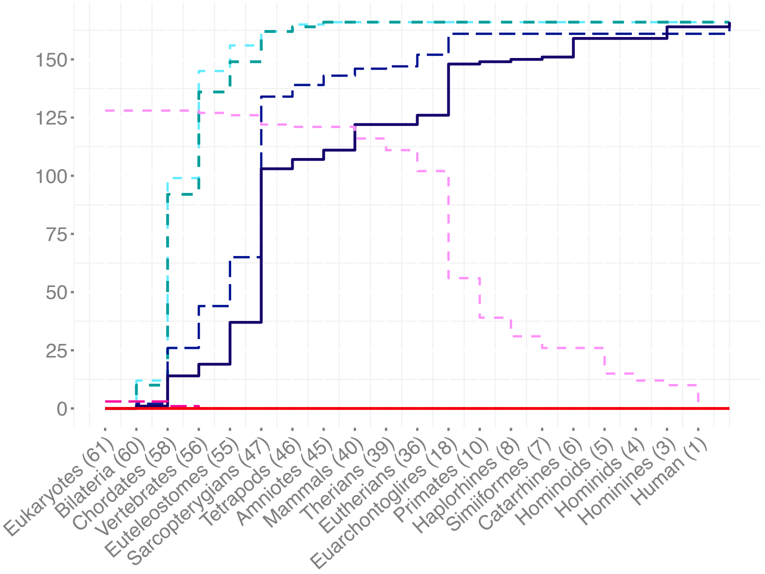
150
125
100
75
50
25
0
Eukaryotes (61)
Bilateria (60)
Chordates (58)
Vertebrates (56)
Euteleostomes (55)
Sarcopterygians (47)
Tetrapods (46)
Amniotes (45)
Mammals (40)
Therians (39)
Eutherians (36)
Euarchontoglires (18)
Primates (10)
Haplorhines (8)
Simiiformes (7)
Catarrhines (6)
Hominoids (5)
Hominids (4)
Homininae (3)
Human (1)

E **Cadherin**

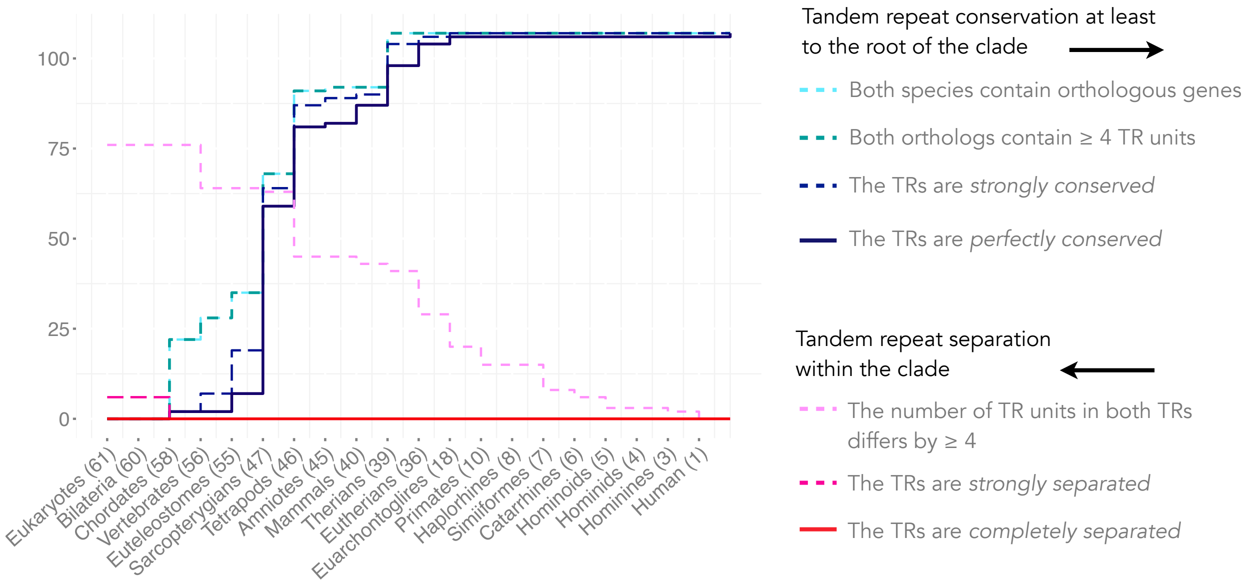

F **I-Set**

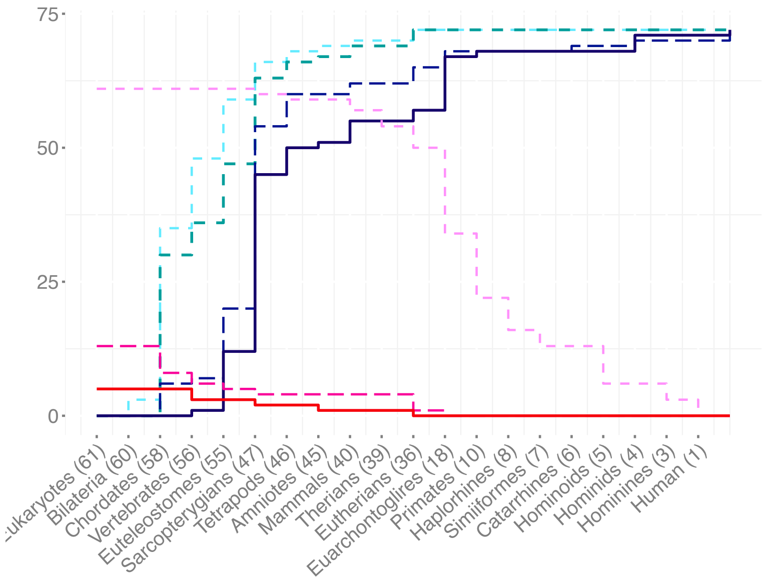

S6 Substitution rates in TRs

		All	Zn finger	LRR	WD40	ANK	*de novo*
All	d_{Flanks}	0.76	2.17	0.43	0.33	0.48	1.18
	$d_{\text{TR region}}$	0.36	0.54	0.21	0.09	0.14	1.41
Strongly conserved	d_{Flanks}	0.28	0.29	0.25	0.33	0.27	0.17
	$d_{\text{TR region}}$	0.12	0.04	0.10	0.08	0.12	0.09
Strongly separated	d_{Flanks}	3.50	4.10	-	0.67	-	4.24
	$d_{\text{TR region}}$	1.20	1.13	-	0.40	-	1.75

Suppl. Table S6: **Average substitution rates per site in the TR region and the TR flanking sequence for the most common TR types (see Material & Methods).**

References

Chang, M. S. S. and Benner, S. A. (2004). Empirical analysis of protein insertions and deletions determining parameters for the correct placement of gaps in protein sequence alignments. *Journal of molecular biology*, 341(2):617–631.

Katoh, K. and Toh, H. (2008). Recent developments in the MAFFT multiple sequence alignment program. *Briefings in bioinformatics.*

Le, S. Q. and Gascuel, O. (2008). An improved general amino acid replacement matrix. *Molecular biology and evolution*, 25(7):1307–1320.

Schaper, E., Kajava, A. V., Hauser, A., and Anisimova, M. (2012). Repeat or not repeat?–Statistical validation of tandem repeat prediction in genomic sequences. *Nucleic acids research*, 40(20).
